# The value of home-based advance care planning in addressing existential concerns among older Norwegian patients with cancer and their relatives: A narrative ethnographic study

**DOI:** 10.1177/26323524251330658

**Published:** 2025-04-21

**Authors:** Line Elida Festvåg, Beate Lie Sverre, Ørnulf Paulsen, Grethe Eilertsen

**Affiliations:** 1 USN Research Group of Older Peoples’ Health, Faculty of Health and Social Sciences, University of South-Eastern Norway, Drammen, Norway; 2 Department of Nursing and Health Sciences, Faculty of Health and Social Sciences, University of South-Eastern Norway, Drammen, Norway; 3 Palliative Care Unit, Telemark Hospital Trust, Skien, Norway; 4 European Palliative Care Research Centre (PRC), Department of Oncology, Oslo University Hospital, Norway; 5 Institute of Clinical Medicine, University of Oslo, Norway

**Keywords:** home-based advance care planning, existential concerns, palliative care, older adults, narrative ethnography

## Abstract

**Background:**

The importance of holistic and person-centered palliative care is widely recognized, but the existential dimensions remain less focused. There is a need for more knowledge about the existential content in advance care planning (ACP) and the significance thereof in the context of home-based palliative care from the perspective of patients and their relatives.

**Objective:**

This study aimed to explore home-based ACP as an existential conversation when conducted as a part of a palliative-care coordination meeting, and how older home-dwelling patients with advanced cancer and their relatives experience its significance.

**Design:**

A narrative ethnographic design was employed.

**Methods:**

The reported empirical material is based on participant observations of the home-based ACP conversations and follow-up interviews with eight patients and their relatives. The analytical approach was guided by the theoretical framework of narrative ethnography.

**Results:**

The ACP conversations strongly supported the patients and their relatives in discussing existential concerns, such as existential fear, lived lives, and decision-making at the end of life. The significance of these conversations extended beyond the initial conversation by profoundly influencing subsequent family interactions and enhancing their ability to make informed and meaningful decisions regarding future medical treatment and care.

**Conclusion:**

Home-based ACP can be a valuable approach for addressing the existential concerns of older patients and their relatives in the final stages of life. Home-based ACP conversations start a process that continues within the family context and affects their relational dynamics and communication patterns. This study indicates that the home environment fosters discussions about past, present, and future lives, shaping the content and outcomes of home-based ACP.

## Introduction

The decision by healthcare professionals not to offer or to discontinue anticancer treatment can evoke significant distress among patients and their relatives and lead to existential concerns.^1,2^ This article addresses the multifaceted landscape of advance care planning (ACP) within the context of such experiences. ACP allows patients with decisional capacity to identify their values, reflect on serious illness scenarios, set goals for their future care, and discuss these aspects with family members and healthcare providers.^3^ ACP is often defined as focusing on patients’ values and preferences regarding medical care and treatment.^4^ However, in our study, we adopt a broader and more comprehensive understanding of ACP, consistent with the definition presented by Rietjens et al.^3^: “ACP addresses individuals’ concerns across the physical, psychological, social, and spiritual domains.” This perspective aligns closely with the holistic approach of palliative care. ACP plays a pivotal role in high-quality palliative care, with numerous studies highlighting its significant benefits, including enhancing communication, the quality of care, and the likelihood of patients spending their final days in their preferred environments.^1,5,6^ Home-based palliative care is widely accessible in Norway and is a standard option for people with advanced illnesses, especially those who wish to stay at home in the final stages of life. Tjernberg and Bökberg^7^ emphasized how important it is for older adults to express their concerns and to discuss essential end-of-life issues to improve their well-being. Older patients with cancer frequently agree with the recommendations of oncologists,^8^ but they also wish to participate in the decision-making process^7,9^ and to be involved in aspects other than simply deciding about their medical options.^10^ However, older home-dwelling patients with advanced cancer only occasionally participate in such discussions.^11,12^ Furthermore, the uptake of ACP is generally low, and this tends to occur late in the disease trajectory, particularly among patients in private environments in comparison with those residing in institutions.^13,14^ This situation underscores the need to improve the communication process and the accessibility of home-based ACP at the end of life.

People with advanced cancer become profoundly more conscious of their mortality.^2^ However, existential dimensions remain the least developed and neglected dimension of palliative care.^15–17^ Nygaard et al.^18^ considered that there is currently no clarity or uniformity in understanding the “existential” concept, and in the present study we relied on their definition:

The term “existential” related to health refers to the fundamental, basic condition of being a human. The existential is based on the irrefutable fact that we live and will die, facing conditions and uncertainties beyond our control. The existential is expressed primarily through a quest for making and seeking meaning in life in general and in demanding life situations. This may involve movements between suffering and re-orientation and meaning and meaninglessness. Existential concerns can be integrated into religious, secular, and spiritual world views.

Yalom^2^ argued that several factors are fundamental to understanding existential concerns: the unavoidable nature of *death* and the desire to continue living, the *freedom* to make decisions to give structure to life, the striving for connection and security despite *existential isolation*, and the quest to find meaning in a *meaninglessness* world. The existential experience in advanced cancer involves mortality and an awareness of death,^19^ underlining the significance of considering the patient’s existential needs when planning future palliative care.

The relatives of a patient are also affected by the shift from active anticancer treatment to end-of-life care. Home-based palliative care has notable benefits,^20,21^ but it also imposes significant burdens on family caregivers, who often shoulder the responsibilities of specialized care alongside healthcare professionals.^22^ Being close to a beloved person facing death may induce existential concerns in relatives. Relatives in this situation often experience a fundamental disruption of reality and need to reframe their perception of normalcy to preserve their self-identity and hope, enhance their self-efficacy in difficult situations as well as meaning in their lives, and prepare for the impending loss of their loved one.^23^ Although relatives are often somewhat invisible, recognizing their pivotal role in this setting is imperative to providing them with adequate support and recognition to alleviate the challenges they face.^24,25^

Binder^26^ argued that while existential suffering is often seen as a psychological deficit in Western culture, it is an unavoidable aspect of human life that must be faced rather than avoided. Consistent with this perspective, Tarbi and Meghani^19^ highlighted the importance of not only acknowledging and addressing existential suffering but also promoting existential health. Patients might prefer to discuss and confront such concerns with their relatives rather than with healthcare professionals due to their private and intimate nature.^27^

ACP conversations often prove complex and challenging due to barriers faced from various sources, such as the reluctance of patients and their families to engage, and inadequacies of the healthcare system such as the inconsistent delivery of promised benefits.^14,28,29^ Although ACP has been studied widely, there is a noticeable gap in the discourse regarding the perspectives of patients and their relatives on ACP as an existential conversation. Furthermore, research into how existential concerns is communicated in the clinical setting remains scarce.^30^

This study formed part of a research project investigating how older patients perceive the significance of ACP conversations in the home. Analyses of the collected data revealed that the conversations held considerable existential significance. Thus, the present study aimed to explore home-based ACP as an existential conversation and how older home-dwelling patients with advanced cancer and their relatives experience its significance. The following research questions were addressed:

1. Which existential issues are addressed during ACP conversations, and how are they addressed?
2. How do patients and their relatives experience the significance of ACP conversations in the home in relation to existential concerns, both during and after the conversations?
3. What characterizes the existential dialog within families after the ACP conversation?

## Methods

### Study design

A narrative, ethnographic approach was chosen to capture the complexity of ACP in home-based palliative care as this approach is well suited to examining narratives within their specific cultural and social contexts.^31,32^

### Setting

This study was conducted in a county with both rural and urban communities. Norway follows a Nordic welfare model where the right to healthcare services is determined by law.^33^ Most healthcare services are public and financed through taxes, ensuring universal access to health services for all citizens regardless of their income. The regional trust is responsible for specialist healthcare services, including hospitals that provide acute diagnostics, medical treatment, and palliative care.^34^ Home-based palliative care is provided by general practitioners (GPs), home-based care services, and municipal cancer nurses (henceforth referred to as cancer nurses). These providers are supported by a hospital-based ambulant palliative care team (PCT), which comprises one nurse and one physician. Home-based palliative care is provided by GPs, home-based care services, and municipal cancer nurses (henceforth referred to simply as cancer nurses), supported by the hospital-based ambulant palliative care team (PCT) comprising one nurse and one physician. The PCT operates from a public, medium-sized hospital trust serving a county with 180,000 residents. In this county, a recently implemented practice ensures that all patients are routinely transferred to primary care services when anticancer therapy is discontinued. A coordination meeting is arranged to initiate and coordinate palliative care, preferably in the patient’s home, and includes the patient and their relatives, the GP, the cancer nurse, and the PCT. To establish ACP as a permanent feature, these meetings were expanded to include an ACP conversation. A description of the preparation for ACP and its conduction has been previously reported.^35^ A patient information brochure with questions related to ACP issues was given to the patients before the meetings (Table 1).

**Table 1.** Patient information brochure about ACP.

1. What is your view and understanding of your disease? 2. Do you have thoughts about your current and future conditions? 3. What gives you strength or joy? 4. Is anything worrying you? 5. Regarding your relatives, what are their resources and needs? 6. If you get into a situation where you can no longer make decisions yourself, who do you want to be your decision-maker? 7. Do you have thoughts about the end of life and death? 8. Is there anything else you would like to discuss?

ACP, advance care planning.

### Recruitment and participants

The PCT and cancer nurses assessed eligible home-dwelling patients aged ⩾65 years with advanced cancer and ECOG Performance Status (PS) scores of 0–3^36^ who either had recently discontinued or were not eligible for anticancer treatment. The ECOG PS score assesses the patient’s ability to perform daily activities, and ranges from 0 (fully active) to 5 (deceased). Inclusion and exclusion criteria are presented in Table 2. Subjects were purposively invited in a face-to-face manner to participate by their cancer nurse. No relationships between the researchers and the participants were established before study commencement except between the PCT and the researchers.

**Table 2.** Inclusion and exclusion criteria.

Inclusion criteria	
Age	⩾65 years
Gender	Male or female
Medical care	Referred to PCT
Living situation	Own home
Residence location	Urban or rural
ACP in the home	Agreeing to participate in ACP conversations with a cancer nurse, GP, and PCT
Language	Fluent in written and spoken Norwegian
Consent	Able to provide informed consent to participate
Relatives	Patient agreed to eligible relatives being invited to participate
Exclusion criterion	
ECOG PS score of ⩾4	Patients assessed as completely disabled or confined to a bed or chair

ACP, advance care planning; GP, general practitioner; PCT, palliative care team; PS, performance status.

We aspired to gain deep insights into the experiences of a strategically selected group of patients and their relatives. The initial phase of the analysis began during data generation, and the recruitment process was continually assessed to determine whether the sample provided sufficiently rich, varied, and detailed data to answer the research questions. This ongoing process allowed for a flexible approach, ensuring that we remained focused on the aim and research questions rather than the sample size. The final sample comprised 8 patients and their 16 relatives (10 daughters, 2 sons, and 4 spouses). Based on the data from the included participants, we concluded that this sample was sufficient to achieve the study’s aim.

The characteristics of the participants are presented in Table 3. The participants had vocational or higher education, reflecting a socioeconomically stable and resourceful group typical of the Norwegian middle class. Three patients were either widowers or divorcees. The median time from the ACP conversation to the patient’s death was 63 days (mean: 103 days, range: 16–272 days). By the time of the second interview, three patients had died, and one was too ill to participate. In these families, the relatives’ accounts of the patient’s final days were used to assess their experience. Before the initiation of any study procedures, written informed consent was obtained from all participants. The participants were informed that the study was part of the PhD education of the first author, who is a female nurse specializing in palliative care.

**Table 3.** Characteristics of patients at the time of the ACP conversations (boldface indicates the patients included in the storylines).

Patient (fictitious names)	Age, years	Cancer diagnosis	Time since diagnosis	Time since anticancer therapy ended	ECOG PS score	Living condition	Religious affiliation	Home-care services per day	Education
Alf	79	Lung	2 years	6 weeks	1	Lives alone, son and daughter nearby	CN	3–4	HE
Birger	88	Prostate	11 years	3 years	1	Lives alone, daughter nearby	PC	2	HE
**Cathrine**	**83**	**Colon**	**1 year**	**8 weeks**	**1**	**Lives with husband, daughters nearby and far away**	**ELFC**	**None**	HE
**Didrik**	**78**	**Prostate**	**17 years**	**13 weeks**	**2**	**Lives with wife, no children**	**CN**	**1**	VE
Else	82	Pancreas	3 months	Not received	3	Lives with husband, two daughters nearby	CN	4	VE
**Finn**	**71**	**Kidney**	**2 years**	**2 weeks**	**3**	**Lives with wife and daughter**	**CN**	**3**	HE
Guri	92	Blood	2 months	Not received	3	Lives with husband, sons and daughter nearby	CN	1 per week	VE
Hannah	82	Uterus	2 years	9 months	3	Lives alone, daughter nearby	CN	1	VE

ACP, advance care planning; CN, Church of Norway; ELFC, Evangelical Lutheran Free Church; HE, Higher education (bachelor’s or higher); PC, Pentecostal Church; PS, performance status; VE, Vocational education at the upper secondary level.

### Participant observations and ethnographic interviews

The fieldwork took place between autumn 2021 and autumn 2022 and consisted of participant observations of the ACP conversations and ethnographic interviews with patients and their relatives at two time points: (1) within 1 week and (2) 1 month after the ACP conversation. The first author conducted interviews and observations in patients’ homes while remaining mindful that their comfort when discussing sensitive topics would influence the conversations.

Observation and interview guidelines were developed for this study to explore various aspects of the home-based ACP (Supplemental Material). A reference group consisting of representatives with experience as patients, relatives, and healthcare professionals was established to assist in developing guidelines, analyzing and discussing the empirical material. Examples of issues guiding the interviews were their experiences of preparing for and participating in the ACP conversation in the home; how they perceived the discussion of their preferences, values, and needs, and their sense of involvement; how the conversation addressed the needs of the relatives; and how they perceived that the conversation and any potential agreements were followed up. Probing and clarifying questions were used to elicit rich and detailed responses from the participants. The interview guideline was first tested on one relative to assess its clarity and alignment with the study’s objectives. No further pilot testing was performed since the first test met the research team’s expectations and did not raise any significant concerns. While the initial guidelines provided a framework for interviews, prior interviews informed the content and approach of later interviews, responding to evolving data. The ethnographic descriptions consisted of field notes (comprising 850 pages) from observations of the ACP conversations, reflection notes, as well as transcriptions of the audio-recorded ACP conversations and ethnographic interviews. The mean duration of both the ACP and follow-up interviews was 60 min.

### Narrative analysis

The study applied iterative analysis to inform the ongoing conduct of the fieldwork. The ethnographic descriptions were systematically analyzed. A narrative approach implies using the ethnographic descriptions to construct narratives that function as the unit of further analysis.^32,37^ According to Riessman,^38^ thematic narrative analysis emphasizes “the told” rather than “how” or “why” it is told, focusing on identifying and analyzing the themes or main messages within the narratives. First, all interviews were coded by L.E.F. and G.E., categorized, and grouped by theme. Existential themes emerged as essential in this initial analysis, prompting a deeper look at ACP as an existential conversation. Second, inspired by Yalom^2^ and Binder,^26^ the field notes were reviewed for existential issues guided by the following analytical question: “Which existential issues were addressed during the ACP conversations, and how were they addressed?” Finally, the interviews were reanalyzed to explore how the patients and their relatives experienced *the significance* of the home-based ACP conversation with a focus on existential concerns, both during and after the ACP conversation. The following main plot was constructed: “ACP in the home created a space conducive to discussing existential concerns with the older patients and their relatives.” A narrative analysis inspired by Riessman^38^ guided the process of constructing representative narratives. Based on the plot, three existential concerns emerged prominently from the data. These concerns served as the foundation for constructing three storylines that were structured according to a temporal perspective, as suggested by Riessman.^38^ The patients Didrik, Cathrine, and Finn (all names are fictional) along with their relatives are central to the three storylines since they are representative of distinct patterns that were present in the empirical data (Table 4). Their quotes that encapsulate core findings have been shortened, cleaned of dialect and filler words, and italicized. The experiences of the remaining five patients, while not forming independent storylines, were integrated into the broader analysis to enrich and refine the primary storylines. Personal characteristics have been changed to protect the participants’ identities. Relatives are identified by their relationships with the patients. The term “physician” refers to the PCT physician.

**Table 4.** The participants present during the ACP conversations for the three families presented in the narratives.*

Patient	Spouse	Adult children	Cancer nurse	GP	PCT: physician and nurse		Researcher
Cathrine	Chris	Camilla, Cate	x	x	x	x	x
Didrik	Dina	-	x	x		x	x
Finn	Frida	Freja	x	x	x		x

* The remaining five ACP conversations had nearly identical attendance distributions.

ACP, advance care planning; GP, general practitioner; PCT, palliative care team.

### Ethics

This study was approved by the Norwegian Centre for Research Data (Reg. no. 316723) in accordance with Norwegian research law and regulations. Two interviews were postponed due to the health of specific patients deteriorating. During the interviews, there was a focus on balancing the obtaining of valuable data while showing respect without being overly cautious.

## Results

Our overarching findings can be summed up by the plot of “ACP in the home created a space conducive to discussing existential concerns with the older patients and their relatives.” To underpin this plot, three storylines are presented: (1) creating space to deal with existential fear, (2) creating space for stories of lived life, and (3) creating space for decision-making at the end of life.

### Creating space to deal with existential fear

Didrik is married to Dina. They have no children, but they have a large family and good friends. Didrik was diagnosed with prostate cancer 15 years ago, and the cancer has metastasized to his bones within the past year. He has undergone extensive chemotherapy, which was stopped 3 months ago. Didrik recounts: “*At the hospital, I felt well taken care of. But now there is nothing more to be done. I am going to die. What scares me most is the constant pain*.” Dina has suffered from severe headaches and poor sleep: “*It’s demanding. But what is even worse, is to see him in pain*.” During his working life, Didrik was used to planning and taking responsibility. When he became ill, they sold their old house to ensure that Dina had a future with fewer practical burdens: “*But what will happen now?*” he wonders, and continues, “*It’s painful to imagine that Dina will be left alone. How will she manage?*”

To discontinue anticancer treatment activates existential concerns as the patient realizes that death is approaching. Like several of the patients, Didrik conveys that he gradually developed a trust in the hospital’s staff about his cancer treatment. However, he now feels his situation is less predictable. The existential fear of being left to fend for themselves is significant for both Didrik and Dina and is expressed when we enter their home.

As we open the front door, Dina welcomes us with a smile and a warm handshake. Didrik stands behind her, and it is clear that his body is frail. The table in the living room is set for six, but Didrik suddenly grabs the sleeve of the GP (who he knows well) and says, “*What is going to happen now? I have been taken out of the system (the hospital, my note). They simply threw me out. I am at a crossroads and don’t know who is responsible. Who will discover that I need a blood transfusion or am in pain?*” He looks at his GP, then at the physician, and back again, raising his voice: “*Are you just going to check if I’m breathing and then leave me here, lying in a corner?*” The physician responds: “*Let’s sit down, Didrik. We’ll do our best to answer all your questions.*” Didrik and Dina sit next to each other. The GP, the cancer nurse, the physician, and I (the first author) take our places around the table. The physician explains that from now on the municipal healthcare service has the main responsibility for Didrik. Didrik is given a list of phone numbers for the people they can call if needed. The physician asks, “*What worries you, Didrik?*” Didrik responds that the pain is severe and that he hesitates to take the small blue pills (opioids). The physician says, “*Didrik, there is still a lot we can do for you even though you are not receiving chemotherapy anymore. The goal is to provide you both with what you need here at home.*” Didrik replies, “*Fine, but you mustn’t slip up.*” The physician emphasizes that if Didrik benefits from blood transfusions, he will receive them, and pain medications will be adjusted as his needs change: “*We (PCT) will come back to visit you when needed. When you become sicker and weaker, we may need to talk more about the things we’ve touched on today, such as if a potential infection should be treated or not.*” The physician turns to Dina: “*Your health is important. You should not pay a too high price for Didrik to be at home.*”

Didrik’s and Dina’s existential concerns were addressed during the ACP conversation when the healthcare professionals visited their home. When we meet one week later, Didrik expresses relief over the reassurances from the physician during the ACP conversation: “*It was a good meeting. We are not thrown into the void. They will help me reach the end in the best possible way.*” Dina adds: “*The most important thing is knowing that we will be taken care of in the time ahead. Now we know who to contact.*”

Four weeks after the ACP conversation, Didrik and Dina report feeling cared for despite Didrik’s severe pain: “*He still hesitates to take the pain medication*,” says Dina, who admits she is tired. Didrik is now much weaker than when we last met, but he smiles and acknowledges, “*Yes, I am still learning. Now I’m waiting for a stay at the hospice unit at the nursing home for a better pain assessment.*”

### Creating space for stories of lived life

Cathrine is in her early eighties and lives with her husband, Chris. Their twin daughters live nearby with their families and are in daily contact with their parents. Cathrine’s health was good until she was diagnosed with advanced cancer 1 year ago. When the hospital physician suggested that it was time to stop anticancer treatment 8 weeks ago, she felt both sorrow and relief. The relief was tied to the unbearable side effects of the treatment: “*The side effects of the last treatment were horrible. I hoped the doctor would say ‘enough is enough*,’ *and he did*,” Cathrine explains. Her cancer had metastasized, and she agreed to the ACP conversation.

Cathrine greets us with smiling eyes and a firm handshake. She looks vital and healthy despite the severity of her cancer. Since the treatment ended, she has not noticed significant changes in her daily life, and she struggles to grasp that she is seriously ill. She says, “*It’s hard to accept that I’m sick. I take care of the house and garden as before.*” The setting includes a piano, pictures of children, wedding photos, and a view of the garden with beehives and fruit trees. We sit in the family’s living room: Cathrine, Chris, their two daughters, her cancer nurse, her GP, and the PCT team. The physician asks as a part of the ACP conversation, “*What gives you strength*?.” Cathrine replies, “*Well, we are still active and do practical things for both children and grandchildren.*” She then speaks of good days and the care she has received from her children, in-laws, and grandchildren. Cathrine describes a fulfilling life with close family ties: “*We moved abroad with small children and stayed there until they started high school. My husband and I worked a lot and took turns looking after the children. It was a good time with hard work from early morning to late at night*.”

Cathrine and her husband, who is also over 80 years old, still appreciate keeping their family home in good order. Her descriptions are strongly rooted in everyday life, which she says gives her the energy to bake, clean, and tend the garden. At the same time, she is well aware of what lies ahead: “*The day will come soon enough when I can’t do anything anymore. For now, we take one day at a time and prefer not to worry about tomorrow*.” Chris agrees and repeats, “*We take one day at a time and enjoy it.*” He is quietly present, affirming without many words, acknowledging, and respecting her portrayal of life before and now, and how they want it to be.

During the conversation, Cathrine and her relatives tell stories of their lived lives that help the healthcare professionals to get to know them, and which convey their values and what they find existentially meaningful. During the subsequent interview, Cathrine’s daughters express a sense of existential validation in the ACP conversation, which extends beyond the mere recognition of her medical condition. Cathrine feels acknowledged for who they are and the life they live: “*Mom was met wonderfully. The healthcare professionals asked about our family and saw her as a mother, grandmother, and professional woman with a full life behind her.*” Cathrine’s husband Chris also highlights his experience of being recognized during the conversation: “*We were met with respect and included. I don’t feel sidelined.*”

Four weeks after the ACP conversation, Cathrine’s health remains unchanged, and she still speaks of good days: “*We live more in the moment and enjoy the family and what’s happening.*” Her family describes how the ACP conversation influenced their relationship and contributed to deeper and more-existential conversations among their family. Camilla describes it this way: “*I see ACP as a ‘rite de passage,’ a marker and aid to understanding that we have entered a new and unknown phase. It helps us to find footing in the new. It was a reality check. The topics we discussed are easier to bring up more frequently again. Our conversations are deeper, and it has affected the level of intimacy between us. It feels good.*” Her sister confirms this experience and adds, “*No matter what happens, I know we stand together.*” Their daughters directly link the sense of unity and intimacy to the ACP conversation.

### Creating space for decision-making at the end of life

Finn is in his early seventies and lives with his wife, Frida. He was diagnosed with renal cancer 2 years ago, and his anticancer treatment was discontinued 2 weeks before the ACP conversation. His worsening general condition, speech difficulties, and nutritional problems led to the healthcare professionals offering Finn and his family an ACP conversation, despite their awareness of his reluctance to discuss the future. In addition to Finn and Frida, their youngest daughter Freja, the cancer nurse, the GP, and the physician participate in the ACP conversation.

We sit outside on the cool veranda, with Finn flanked by his wife on one side and his daughter on the other: “*I feel trapped in a body that no longer works.*” He pauses briefly before continuing, “*I need help with everything and feel guilty that my wife has to do what once was my responsibility*.” He nods toward the ladder leaning against the freshly painted wall, takes a breath, and says pointedly, “*But I don’t like talking about it*.” The conversation gradually shifts to questions about life-prolonging treatment. The physician asks gently, “*We would like to know if you have any thoughts about this, such as treatment for infections or hospitalization. Have you thought about it?*” Finn looks uncomfortable: “*I don’t like talking about these things*.” His wife places a hand on his arm and elaborates, “*He prefers to avoid such conversations and often jokes about it. I respect that. When the time comes, I believe he will answer. Not now.*” Their daughter nods in agreement, “*We prefer not to talk about it*.” After a pause, the physician ventures further: “*What worries you, Finn?*” Finn looks down at his folded hands and replies that his biggest concern is the pain: “*My mother died of cancer and had a lot of pain. I am worried about the pain.*” The physician continues by explaining the potential worsening of the illness, palliative care, and the importance of knowing the patient’s preferences: “*We take responsibility for assessing what is best for you. You will not be offered futile hospitalizations or treatments that could cause you significant distress. However, we need to know if you have any thoughts about the options for hospitalization and antibiotic treatment in case of infections. But you don’t need to make these decisions today*.” Finn nods.

This part of the narrative underscores the difficulty of discussing future medical treatment and care at the end of life. Finn’s reaction indicates his great uncertainty when he is asked existential questions related to his autonomy.

When I (the first author) visit Finn, his wife, and their daughter 1 week later, he is sitting on the veranda with his eyes closed, listening to music. He smiles and slowly stands when he notices me. Shortly thereafter he admits that he felt uncomfortable when discussing what lies ahead in the ACP conversation: “*I couldn’t bring myself to read the information leaflet about ACP before they came. I take one day at a time.*” Frida looks at her husband and says, “*But we have an agreement that you will stay home as long as possible.*” She says that she works in healthcare herself and is well aware of what the future will require from her: “*Our daughter has moved home for a period.*” Their daughter Freja, who has been listening to her parents’ story, now says, “*I think neither Dad nor we understood what this conversation entailed. If it had been discussed before the actual conversation, I think we could have delved deeper into our thoughts.*”

Finn died 3 weeks after the ACP conversation. His relatives still wished to participate in the second interview as they were eager to describe how ACP had probably helped Finn to convey his preferences on the day when his condition suddenly deteriorated. Freja recounts, “*A few days after ACP, he developed pneumonia. This time, Dad was clear about what he wanted: treatment at home. But then he fell, which led to hospitalization with concerns about a suspected bone fracture or head injury. Thankfully, there were no injuries. But the attending hospital physician did not want to let him go home because he was ‘too ill.’ When Dad heard that decision, he tried to remove the intravenous needle. I asked him, ‘Do you want to go home, Dad?*’ *and he mimed ‘yes.*’ *I promised Dad that he would come home. Thanks to the interaction between the hospital, PCT and municipal healthcare staff, Dad was discharged the next day and died at home five days later. I believe that even though Dad did not make any decisions during the ACP conversation, that made him understand that he had a choice. When he ended up in the hospital, we were confident in the decision to bring him home. We knew what he wanted*” (visibly moved, tears streaming down her cheeks).

## Discussion

This study has revealed that home-based ACP can open spaces for existential conversations between older patients, their relatives, and healthcare professionals: a space to deal with existential fear, a space for stories of lived life, and a space for decision-making at the end of life.

### Dealing with existential fear

The fear of being left to fend for themselves without necessary healthcare was a *pervasive existential concern*. This fear could be interpreted as the transition involving being disconnected from the hospital after the discontinuation of anticancer treatment. Gardiner^39^ highlighted how patients experience fear and uncertainty during such a transition. Ensuring that patients understand the continuum of care available to them, including palliative-care options, is considered crucial to addressing these existential concerns. Walczak^40^ found that discussing the prognosis and end-of-life care issues, and then putting plans in place enabled patients to put their concerns about the future aside and to focus on their quality of life.

The study setting was ACP conducted during meetings for initiating and coordinating palliative care in the presence of several healthcare professionals. The GP and the cancer nurse were familiar and trusted persons, and hence represented continuity that encouraged openness, whereas the other healthcare professionals were unfamiliar to the patients. Nevertheless, this unfamiliarity did not appear to hinder openness or their expressions of fear, as patients freely voiced their concerns during the ACP conversations. This is noteworthy given that patients are usually reluctant to openly express negative or distressing emotions.^41^ The home setting seemed to facilitate the dynamics between the patients, their relatives, and healthcare professionals. Trust and perceived supportiveness are reported to significantly influence the willingness of patients to discuss sensitive topics, such as end-of-life concerns.^40,42,43^ The home-based ACP conversations in the present study provided opportunities to talk about fears and concerns that had not previously been addressed.

### Maintaining a meaningful life

The *second existential concern* involved the patients’ efforts to maintain a meaningful life. Meaning in life is essential to humans^44^ and involves experiencing life as coherent and purposeful, with a sense of direction and belonging.^45^ Meaning-making of life events and constructing identities is often achieved through storytelling in which individual events are woven into a cohesive and meaningful story.^46^ During the ACP conversations, the patients and their families produced narratives that encompassed life stories, significant relationships, and daily activities. Serious illness and impending death are often perceived as threats to one’s identity, which may lead to questions such as “who have I been?”, “who am I now?”, and “who do I wish to be, and for whom?”.^2,47^ By recounting their life stories, the patients conveyed their identities across time, recognizing roles and experiences, and linking past, present, and future lives, which fostered continuity and coherence amid illness and uncertainty. Valuing these life stories during the ACP conversations established their current situation within a broader, meaningful context, which is especially important when nearing the end of life.

Despite being fully aware of their truncated life span, the patients and their relatives chose to focus on the positive aspects of life, such as everyday tasks (no matter how trivial) that provided strength and joy. This dynamic movement within and between existential suffering and joy has been described as the true existential experience.^19^ Our study found that focusing on daily activities and close relationships helped the patients to navigate their illness and give their lives a sense of meaning. This is consistent with previous research showing that older patients with advanced cancer prioritize independence and daily routines, and value outdoor access and gardens.^48–51^

The patients and their relatives appreciated that the healthcare professionals showed empathy and presence, which promoted their feeling of being acknowledged as persons. The healthcare professionals found it important to have visited the patients in their homes, since this facilitates the recognition and appreciation of each person’s uniqueness as well as the communication needed to identify the values, preferences, and needs of patients.^35^

Some patients prefer to discuss and confront death with their relatives rather than with healthcare professionals due to the private and intimate nature of this topic.^27^ One of the daughters in the present study described an existential process by which the siblings were confronted with a new reality and the impermanence of life as they realized that their mother was going to die. The ACP conversation helped to navigate this landscape and facilitated the patients and their relatives being fully present for each other, strengthening their existential closeness. This increased engagement in more-candid discussions about life and the future was a consistent finding across most of the narratives. This demonstrates that the home-based ACP conversations started a process that extended beyond the initial ACP conversation, continuing within the family context and affecting their relational dynamics and communication patterns.

### Decision-making at the end of life

The *third storyline* highlights the significance of the home-based ACP conversation in engaging older patients in discussions regarding their future medical treatment and care. During the ACP conversation, the patients expressed uncertainty about making decisions. As exemplified by Finn, the patients found it difficult to address questions regarding life-prolonging treatment during the ACP conversation. However, the conversation prompted a reflective process within the families. These reflections were further accelerated when Finn’s medical status deteriorated, and he developed a pneumonia. After that, he expressed firm wishes to receive treatment at home and to not be treated at the hospital. This further exemplifies how the process gave his daughter the unique competence and strength to understand and support his father’s wishes, and even to confront the healthcare professionals to make them withdraw the treatment they otherwise would have automatically offered him.

The ability to make choices is a fundamental existential concern.^2^ However, decision-making and self-determination can be particularly challenging for patients at the end of life, who are often vulnerable and reliant on others. Their capacity to make informed decisions can diminish, which impairs their autonomy. One daughter described this as a lack of readiness to engage in such discussions. Moreover, Finn’s reluctance may reflect an adaptive denial strategy or the engagement of an avoidance strategy, which was described by Romo et al.^52^ as patients either accepting the progression of illness without fixating on it or actively avoiding thinking about the end of life. Vandenbogaerde et al.^53^ described that on one hand, patients with amyotrophic lateral sclerosis may resist conversations about end-of-life care and decision-making due to the uncertainty of the illness trajectory, to protect themselves and their relatives from distressing emotions and to remain stable. On the other hand, when their condition deteriorates, many of these patients come to recognize the critical importance of these conversations, which motivates them to engage in decision-making before they can no longer express their wishes. Moreover, Houska and Loučka^54^ argued that autonomy should not be seen merely as the ability to make decisions *independently*; rather it should encompass the complexities and contextual nuances associated with living with advanced illness. Our study supports this nuanced understanding of autonomy. Active control in the context of ACP included not only deciding about future scenarios but also assisting patients and their relatives with decision-making as the circumstances became clearer. The existential nature of the ACP conversation proved invaluable in guiding the family’s decision-making process, increasing the abilities of both the patients and their relatives to make end-of-life decisions. This emphasizes the need to raise awareness of the ACP’s purpose and ensure that healthcare professionals have the time and competence to facilitate these conversations. An upcoming sub-study from this project will address this.

To sum up, our study has revealed that home-based ACP, through its relational and communicative dynamics in the context of home, can address existential concerns and facilitate a process of continuing existential conversations within the families as well as person-centered decision-making.

### Strengths and limitations

The ethnographic design of this prospective study enabled a thorough investigation of how existential concerns were addressed in the home-based ACP conversations and described the communication process within the families during the following weeks. Conducting interviews at two different time points provided insights into the evolving significance of home-based ACP as a process from the perspectives of both patients and their relatives.

The first author’s presence during the ACP conversations allowed for a deeper understanding of the dynamics and context when patients and their relatives subsequently shared their experiences, ensuring more accurate and contextually grounded insights. Trustworthiness was ensured through detailed descriptions of the researchers’ backgrounds, study context, and analysis strategy, along with quotes from the participants, a rigorous and consensual interpretation process by the research team comprising multiple professionals, and feedback from a reference group to explore different interpretations of the findings.

One strength of the study was its prospective design that included two follow-up interviews, which allowed observations of continuous interactions within the families. However, one potential limitation was not controlling for situations where one party might withhold concerns to protect the other. The goals of achieving a balanced gender distribution and including patients from both urban and rural areas were achieved. Though, when interpreting the findings, it is essential to be aware of the patient groups excluded from the study, such as older adults with cognitive impairments, patients without family, and those from diverse cultural backgrounds, as they may experience ACP differently. Our findings primarily reflect positive attitudes toward home-based ACP. However, this might have been biased by the ACP conversations being conducted by healthcare professionals with extensive experience in palliative care, communication, and decision-making. Moreover, patients with negative attitudes might have been more likely to refuse to participate. We did not obtain information about the patients who declined to participate.

## Conclusion

This study found that home-based ACP created a conducive space for older patients and their relatives to discuss existential concerns by addressing their existential fears and sharing life stories and end-of-life decisions. The home-based ACP also fostered not only immediate conversations about the existential concerns, but also subsequent discussions within the families. This process provided them with clearer communication and enhanced competence regarding their thoughts and preferences about ongoing treatment, which ultimately strengthened their autonomy. This can significantly influence intrafamily relationships, the quality of patient care, and the overall end-of-life experience for both patients and their relatives.

The study indicates that the home-setting can enhance the effectiveness of ACP.

## Supplemental Material

sj-docx-1-pcr-10.1177_26323524251330658 – Supplemental material for The value of home-based advance care planning in addressing existential concerns among older Norwegian patients with cancer and their relatives: A narrative ethnographic study Supplemental material, sj-docx-1-pcr-10.1177_26323524251330658 for The value of home-based advance care planning in addressing existential concerns among older Norwegian patients with cancer and their relatives: A narrative ethnographic study by Line Elida Festvåg, Beate Lie Sverre, Ørnulf Paulsen and Grethe Eilertsen in Palliative Care and Social Practice

sj-docx-2-pcr-10.1177_26323524251330658 – Supplemental material for The value of home-based advance care planning in addressing existential concerns among older Norwegian patients with cancer and their relatives: A narrative ethnographic study Supplemental material, sj-docx-2-pcr-10.1177_26323524251330658 for The value of home-based advance care planning in addressing existential concerns among older Norwegian patients with cancer and their relatives: A narrative ethnographic study by Line Elida Festvåg, Beate Lie Sverre, Ørnulf Paulsen and Grethe Eilertsen in Palliative Care and Social Practice

sj-docx-3-pcr-10.1177_26323524251330658 – Supplemental material for The value of home-based advance care planning in addressing existential concerns among older Norwegian patients with cancer and their relatives: A narrative ethnographic study Supplemental material, sj-docx-3-pcr-10.1177_26323524251330658 for The value of home-based advance care planning in addressing existential concerns among older Norwegian patients with cancer and their relatives: A narrative ethnographic study by Line Elida Festvåg, Beate Lie Sverre, Ørnulf Paulsen and Grethe Eilertsen in Palliative Care and Social Practice
