# Supplementary material for: The value of home-based advance care planning in addressing existential concerns among older Norwegian patients with cancer and their relatives: A narrative ethnographic study
Source: Palliat Care Soc Pract. 2025 Apr 21;19:26323524251330658. doi: 10.1177/26323524251330658 (PMC12033682; doi:10.1177/26323524251330658)
Supplement: sj-docx-2-pcr-10.1177_26323524251330658 – Supplemental material for The value of home-based advance care planning in addressing existential concerns among older Norwegian patients with cancer and their relatives: A narrative ethnographic study [file sj-docx-2-pcr-10.1177_26323524251330658.docx]

**Interview guide with patients and relatives after the advance care planning (ACP) conversation**

**First Interview Shortly After the ACP Conversation:**

***Primarily for the Patient***

1. How did you experience preparing for the conversation?

i. What information did you receive about the ACP conversation beforehand?

ii. Do you feel this information helped you in your preparation?

iii. Are there questions in the information brochure that you find more important than others?

iv. Are there any questions you feel are missing from the information brochure? Which ones?

v. What considerations did you consider when deciding to agree to the ACP conversation?

**For Relatives:** What thoughts and experiences do you have regarding what we have discussed now?

2. How did you experience participating in an ACP conversation in your own home?

i. What was important for you to discuss and clarify during this conversation?

ii. How did you feel to talk about these matters with so many people present (palliative care team, general practitioner, and cancer nurse)?

iii. Do you feel that the content of the ACP conversation is significant for you now and in the near future?

iv. Did you find any subjects difficult to discuss? Were there any things you preferred not to talk about during the conversation?

v. Do you feel that you were able to express what is important to you? Were there any questions you would have liked to discuss with the cancer nurse or general practitioner that haven't been addressed yet?

**For Relatives**: What thoughts and experiences do you have regarding what we have discussed now?

3. How do you perceive the significance of the ACP conversation in terms of staying at home as long as possible?

i. Do you feel that ACP conversation can help clarify any misunderstandings or disagreements between you and your relatives?

ii. Are there any questions or topics from the conversation that you have discussed with your relatives afterward? If so, which ones?

**For Relatives**: What thoughts and experiences do you have regarding what we have discussed now?

4. How do you feel that the current plan for treatment and care aligns with your wishes and needs?

i. Do you feel that questions concerning the future (e.g., symptom relief, hospital admission, preferred place of care, potential antibiotic treatment) were clarified?

ii. How do you perceive the opportunity for participation in decisions regarding palliative care and treatment?

**For Relatives**: What thoughts and experiences do you have regarding what we have discussed now?

5. Documentation of the advance care planning conversation.

i. Did you participate in drafting or reviewing the notes from the ACP conversation?

ii. Have you received a copy of the notes from the ACP conversation, or been offered one?

**For Relatives**: What thoughts and experiences do you have regarding what we have discussed now?

**Second Interview 3-4 Weeks After the Advance Care Planning Conversation:**

1. How do you feel the ACP conversation has impacted the time since we last spoke?

i. How do you feel your values, wishes, and needs are being respected and followed up by healthcare professionals?

ii. Can you provide examples of agreements made where you were involved in the content?

**For Relatives**: What thoughts and experiences do you have regarding what we have discussed now?

2. Have there been any changes in needs and wishes since the ACP conversation took place?

i. How do you feel these changes are being followed up by home care services and your general practitioner?

ii. Have there been any new ACP conversations? Who initiated these?

iii. Have there been adjustments to the agreements made during the ACP conversation? Any new agreements?

**For Relatives**: What thoughts and experiences do you have regarding what we have discussed now?
