# Supplementary material for: The value of home-based advance care planning in addressing existential concerns among older Norwegian patients with cancer and their relatives: A narrative ethnographic study
Source: Palliat Care Soc Pract. 2025 Apr 21;19:26323524251330658. doi: 10.1177/26323524251330658 (PMC12033682; doi:10.1177/26323524251330658)
Supplement: sj-docx-3-pcr-10.1177_26323524251330658 – Supplemental material for The value of home-based advance care planning in addressing existential concerns among older Norwegian patients with cancer and their relatives: A narrative ethnographic study [file sj-docx-3-pcr-10.1177_26323524251330658.docx]

**Observation guide for the ACP conversation carried out in the patient’s home**

1. What happens in the ACP conversation?

i. Who participates?

ii. Where does the conversation take place?

iii. When does the conversation take place?

iv. Who takes responsibility for what is discussed during the conversation?

v. What is being talked about?

vi. How do the interactions and communication take place?

vii. How are the participants positioned relative to each other?

viii. How are contextual factors used in the ACP conversation, such as descriptions of the home, pictures, furniture, and garden?

2. What needs and preferences are identified?

3. What agreements are made in the ACP conversation?

i. Agreements and clarifications to be documented in the patient’s medical record, as well as in the transcript of the conversation?

ii. Information provided to the patient about the possibility of corrections, additions, and other changes?

iii. Clarifications about further follow-up of the ACP conversation? Who? When? How?
